# Supplementary material for: Trends in the burden of gastroesophageal reflux disease in China and Global from 1990 to 2021 and predictive analysis for 2040
Source: PLoS One. 2026 Feb 2;21(2):e0341619. doi: 10.1371/journal.pone.0341619 (PMC12863522; doi:10.1371/journal.pone.0341619)
Supplement: Attachment — Submitted filename: 07 07 2025.docx [file pone.0341619.s009.docx]

- The study aims to analyze GERD burden trends in China and globally, but the introduction could better highlight the unique contributions of this work compared to existing literature. Specifically, emphasize how this study addresses gaps in age- and gender-specific analyses and predictive modeling for China, which previous studies have overlooked.
- The use of Joinpoint regression and BAPC models is appropriate, but the manuscript should provide more detail on model validation. For instance, include metrics like goodness-of-fit tests or sensitivity analyses to strengthen the reliability of predictions for 2040.
- The study acknowledges potential biases from GBD data (e.g., underdiagnosis in low-resource regions, masking by PPI use). However, it should discuss how these limitations might affect the comparability of China vs. global trends and suggest ways future studies could mitigate these issues (e.g., regional validation studies).
- The finding that China’s GERD burden declined while global rates rose is intriguing but warrants deeper discussion. Explore potential explanations (e.g., dietary changes, healthcare policies, diagnostic criteria differences) and cite supporting evidence to contextualize these disparities.
- The higher GERD burden in women is noted, but the underlying mechanisms (hormonal, behavioral, or diagnostic biases) are not thoroughly explored. Consider adding a paragraph synthesizing existing hypotheses (e.g., estrogen’s role in LES function) to enrich the discussion.
- The 2040 projections suggest rising incidence/prevalence despite declining DALYs. Clarify this paradox: Is it due to aging populations, improved management reducing disability, or other factors? Link these findings to actionable recommendations (e.g., targeted screening for elderly males).
- Ensure all supplementary tables (e.g., AAPC values, age-stratified rates) are referenced in the main text to support key claims. Figure captions should explicitly state what trends are highlighted (e.g., “ASIR decline in China vs. global increase”).
- The declarations are complete, but confirm that all data sources (e.g., GBD) are properly cited and permissions for reuse are documented, even if publicly available.
- The conclusion could better align with the study’s novel findings. Stress the urgency of gender- and age-specific interventions in China, given the projected rise in male burden, and call for cross-national studies to validate diagnostic disparities.
