# Supplementary material for: Trends in the burden of gastroesophageal reflux disease in China and Global from 1990 to 2021 and predictive analysis for 2040
Source: PLoS One. 2026 Feb 2;21(2):e0341619. doi: 10.1371/journal.pone.0341619 (PMC12863522; doi:10.1371/journal.pone.0341619)
Supplement: Attachment — Submitted filename: Reviewer 2.docx [file pone.0341619.s011.docx]

Note that the following color scheme is used in responding to reviewers’ comments

Black: reviewers’ comments

Blue: our responses

Red: our revisions in the manuscript

To Reviewer 2:

Dear Reviewer 2,

Thank you very much for your thorough review and valuable feedback on our study. We greatly appreciate your suggestions and have made the necessary revisions and improvements to the manuscript. Our specific responses to your comments are as follows:

**Comment 1**:Too dense for journal readership. The abstract exceeds typical word limits and mixes methods with results in a confusing structure.

Please condense the abstract into:Background, Methods, Results (only major findings), Conclusion.

Answer 1:We have carefully re - evaluated the abstract. We have removed any redundant information and restructured it to clearly separate the background, methods, major results, and conclusion. We have also ensured that the word count is within the typical limits for the journal, and we have rewritten the abstract to follow the suggested structure. The background section now provides a concise introduction to the problem we addressed. The methods section briefly outlines the key approaches we used. In the results section, we only include the major findings. Finally, the conclusion summarizes the main implications of our study.(Page 2, Line 22-37)

**Comment 2**:Statistical Clarity:Joinpoint and BAPC methods are referenced well, but readers unfamiliar with these may struggle. Many APC/AAPC values are mentioned without clear context.It's better to move detailed APC results to supplementary tables and add brief, accessible explanations of each method in plain terms.

Answer 2:We have added brief, accessible explanations of the Joinpoint and BAPC methods in plain - language terms within the paper to assist readers who are not familiar with these methods.We have provided more context for the APC/AAPC values in the main text of the paper, clarifying their significance and how they were derived.We have moved the detailed APC results to supplementary tables. Additionally, as mentioned before, we have added plain - language explanations of each method in the main body of the paper.(Page 11, Line 222-236).

**Comment 3**:Statistical Clarity:Language and Grammar:Please use a professional editing service to improve clarity, grammar, and conciseness.

Answer 3: We thoroughly reviewed the manuscript, corrected all grammar errors, refined the sentence structures to improve clarity, and made the text more concise by eliminating redundant words and phrases.

Once again, thank you for your valuable feedback on our research. Your insights not only helped us improve the details of our study but also provided us with precious suggestions for future research directions. We look forward to your further comments.

Sincerely,

[Zexu Tang]
