# Supplementary material for: Trends in the burden of gastroesophageal reflux disease in China and Global from 1990 to 2021 and predictive analysis for 2040
Source: PLoS One. 2026 Feb 2;21(2):e0341619. doi: 10.1371/journal.pone.0341619 (PMC12863522; doi:10.1371/journal.pone.0341619)
Supplement: Attachment — Submitted filename: Editor R2.docx [file pone.0341619.s012.docx]

Note that the following color scheme is used in responding to editors’ comments

Black: editors’ comments

Blue: our responses

To Editors:

Dear Editors,

Thank you very much for your thorough review and valuable feedback on our study. We greatly appreciate your suggestions and have made the necessary revisions and improvements to the manuscript. Our specific responses to your comments are as follows:

Concern 1: Authors should consult with an ethics committee to ensure data are shared in accordance with participant consent and local laws.
Answer: We acknowledge the importance of consulting with an ethics committee and have ensured that our research complies with all applicable local laws and participant consent requirements.

Concern 2: Data sharing should never compromise participant privacy.
Answer: We fully endorse this principle and have implemented necessary measures to protect participant privacy, specifically by anonymizing all potentially identifiable information in the submitted raw data.

Once again, thank you for your valuable feedback on our research. Your insights not only helped us improve the details of our study but also provided us with precious suggestions for future research directions. We look forward to your further comments.

Sincerely,

[Zexu Tang]

Location	ID

Global	1

People's Republic of China	2

Democratic Republic of Timor-Leste	3

Democratic People's Republic of Korea	4

Republic of Maldives	5

Republic of the Philippines	6

Solomon Islands	7

Republic of Kiribati	8

Federated States of Micronesia	9

Republic of Indonesia	10

Kingdom of Thailand	11

Malaysia	12

Independent State of Samoa	13

Lao People's Democratic Republic	14

Taiwan (Province of China)	15

Socialist Republic of Viet Nam	16

Georgia	17

Republic of the Union of Myanmar	18

Republic of Vanuatu	19

Republic of Fiji	20

Democratic Socialist Republic of Sri Lanka	21

Kingdom of Cambodia	22

Kyrgyz Republic	23

Kingdom of Tonga	24

Bosnia and Herzegovina	25

Republic of the Marshall Islands	26

Republic of Poland	27

Republic of Tajikistan	28

Republic of Azerbaijan	29

Republic of Serbia	30

Republic of Croatia	31

Republic of Lithuania	32

Japan	33

Republic of Uzbekistan	34

Independent State of Papua New Guinea	35

Russian Federation	36

Republic of Singapore	37

Republic of Slovenia	38

Republic of Kazakhstan	39

Hungary	40

Republic of Armenia	41

Republic of Albania	42

Montenegro	43

Australia	44

Mongolia	45

Republic of Belarus	46

Czech Republic	47

Republic of Bulgaria	48

Brunei Darussalam	49

Kingdom of Belgium	50

Republic of Latvia	51

Romania	52

Republic of Moldova	53

Slovak Republic	54

Turkmenistan	55

North Macedonia	56

Republic of Iceland	57

Republic of Korea	58

Kingdom of Denmark	59

Republic of Austria	60

State of Israel	61

French Republic	62

Ukraine	63

Eastern Republic of Uruguay	64

Kingdom of Spain	65

New Zealand	66

Republic of Estonia	67

Hellenic Republic	68

Grand Duchy of Luxembourg	69

Swiss Confederation	70

Belize	71

Kingdom of the Netherlands	72

Canada	73

Jamaica	74

Republic of Cyprus	75

Commonwealth of Dominica	76

Portuguese Republic	77

Principality of Andorra	78

Antigua and Barbuda	79

Ireland	80

Grenada	81

Republic of Finland	82

Saint Vincent and the Grenadines	83

Republic of Italy	84

Republic of Chile	85

Republic of Haiti	86

Kingdom of Sweden	87

Barbados	88

Federal Republic of Germany	89

Republic of Trinidad and Tobago	90

Republic of Malta	91

Saint Lucia	92

United Kingdom of Great Britain and Northern Ireland	93

Dominican Republic	94

Republic of Nicaragua	95

Republic of Cuba	96

United States of America	97

Republic of Costa Rica	98

Commonwealth of the Bahamas	99

Argentine Republic	100

Plurinational State of Bolivia	101

Bolivarian Republic of Venezuela	102

Republic of Guyana	103

Republic of Suriname	104

Kingdom of Norway	105

State of Libya	106

Republic of Guatemala	107

Republic of Peru	108

Kingdom of Bahrain	109

Federative Republic of Brazil	110

Islamic Republic of Iran	111

Palestine	112

United Arab Emirates	113

United Mexican States	114

Republic of Colombia	115

State of Qatar	116

Republic of Panama	117

Hashemite Kingdom of Jordan	118

Republic of El Salvador	119

Lebanese Republic	120

Republic of India	121

People's Democratic Republic of Algeria	122

Syrian Arab Republic	123

Republic of Paraguay	124

Republic of Turkey	125

Sultanate of Oman	126

Islamic Republic of Afghanistan	127

Arab Republic of Egypt	128

Islamic Republic of Pakistan	129

Kingdom of Morocco	130

Republic of Ecuador	131

Kingdom of Bhutan	132

Republic of Equatorial Guinea	133

Republic of Honduras	134

Republic of Yemen	135

Kingdom of Saudi Arabia	136

Republic of Iraq	137

Federal Democratic Republic of Ethiopia	138

State of Kuwait	139

Central African Republic	140

Republic of Madagascar	141

Union of the Comoros	142

United Republic of Tanzania	143

Republic of Tunisia	144

Federal Democratic Republic of Nepal	145

Republic of South Africa	146

People's Republic of Bangladesh	147

Democratic Republic of the Congo	148

Republic of Zimbabwe	149

Republic of Mauritius	150

State of Eritrea	151

Republic of Zambia	152

Republic of Rwanda	153

Republic of Côte d'Ivoire	154

Republic of Kenya	155

Republic of Benin	156

Republic of Botswana	157

Gabonese Republic	158

Republic of Burundi	159

Republic of Malawi	160

Republic of Ghana	161

Republic of Angola	162

Federal Republic of Somalia	163

Republic of Cameroon	164

Kingdom of Eswatini	165

Islamic Republic of Mauritania	166

Republic of the Congo	167

Republic of Djibouti	168

Republic of Namibia	169

Republic of Uganda	170

Republic of Guinea-Bissau	171

Republic of Mozambique	172

Republic of Chad	173

Federal Republic of Nigeria	174

Kingdom of Lesotho	175

Republic of Senegal	176

Republic of the Gambia	177

Burkina Faso	178

Bermuda	179

Republic of Mali	180

Republic of Nauru	181

Tokelau	182

Togolese Republic	183

Republic of the Niger	184

Greenland	185

Republic of Seychelles	186

Republic of Cabo Verde	187

Republic of Guinea	188

United States Virgin Islands	189

Northern Mariana Islands	190

Republic of Sudan	191

Democratic Republic of Sao Tome and Principe	192

Republic of Liberia	193

Puerto Rico	194

Cook Islands	195

Principality of Monaco	196

Republic of San Marino	197

American Samoa	198

Republic of Niue	199

Republic of Sierra Leone	200

Tuvalu	201

Republic of Palau	202

Guam	203

Saint Kitts and Nevis	204

Republic of South Sudan	205
