# Supplementary material for: Trends in the burden of gastroesophageal reflux disease in China and Global from 1990 to 2021 and predictive analysis for 2040
Source: PLoS One. 2026 Feb 2;21(2):e0341619. doi: 10.1371/journal.pone.0341619 (PMC12863522; doi:10.1371/journal.pone.0341619)
Supplement: Attachment — Submitted filename: Reviewer 4.docx [file pone.0341619.s013.docx]

Note that the following color scheme is used in responding to reviewers’ comments

Black: reviewers’ comments

Blue: our responses

Red: our revisions in the manuscript

To Reviewer 4:

Dear Reviewer 4,

Thank you very much for your thorough review and valuable feedback on our study. We sincerely thank you for your insightful and constructive comments, which have greatly helped us to improve the clarity and clinical relevance of our manuscript. We have carefully revised the manuscript accordingly. Our detailed responses are provided below:

**Comment 1**:Define uncertainty intervals (UI) clearly in the methodology section.

Answer 1:Thank you for this valuable suggestion. We have revised the Statistical Analysis subsection of the Materials and Methods to provide a clear and explicit definition of uncertainty intervals (UIs). Specifically, we now clarify that all estimates were generated from 500 posterior draws produced by the DisMod-MR 2.1 model and that 95% UIs were defined as the 2.5th and 97.5th percentiles of these draws. We also explain that UIs reflect combined uncertainty from data sources, model structure, and parameter estimation. These revisions improve methodological transparency and consistency with GBD reporting standards.(Page 7, Line 140-147)

**Comment 2**:Can you mention how the lack of age- and gender-specific trends impede the management of refractory GERD (R-GERD)?

Answer 2:Thank you for highlighting this important clinical and public health issue. In response, we have added a dedicated paragraph in the Discussion explicitly addressing how the absence of clearly defined age- and sex-specific epidemiological trends limits the effective management of refractory GERD (R-GERD).

In the revised text, we emphasize that R-GERD is a heterogeneous condition influenced by hormonal status, visceral sensitivity, psychosocial stress, and age-related physiological changes. We further explain that insufficient demographic stratification at the population level may hinder early identification of high-risk subgroups, delay treatment optimization, and constrain the development of individualized management strategies. These revisions clarify the direct link between epidemiological granularity and clinical decision-making in R-GERD management.(Page 16 and 17, Line 365-374)

**Comment 3**:The significant national difference in peak age (China 50s vs. Global 30s) should be addressed in the discussion.

Answer 3: We appreciate this valuable suggestion. We have substantially expanded the Discussion to explicitly examine the marked difference in peak age of GERD burden between China and the global population. The revised manuscript now includes a focused paragraph comparing the later peak observed in China (50–59 years) with the earlier global peak (30–39 years).

In this section, we discuss potential explanations for this discrepancy, including differences in population aging trajectories, cumulative exposure to risk factors, lifestyle transitions, dietary patterns, and healthcare-seeking behaviors. We further highlight that these divergent age patterns may reflect distinct dominant GERD phenotypes across populations, with important implications for prevention strategies and clinical management. This addition strengthens the contextual interpretation of our findings and underscores the need for region-specific approaches.(Page 15, Line 328-340)

.

**Comment 4**:I can’t find out how the data was handled, including imputation and missing data.

Answer 4:Thank you for pointing out the need for greater methodological clarity. We have revised the Methods section to better describe how data uncertainty and missing data were handled within the GBD 2021 framework. Specifically, we now clarify that all estimates were derived using standardized GBD modeling approaches, including DisMod-MR, which integrates multiple data sources and addresses data sparsity and missingness through Bayesian meta-regression.

We further explain that uncertainty intervals were generated from 500 posterior draws, reflecting uncertainty arising from data availability, model specification, and parameter estimation. By explicitly referencing these established GBD procedures and directing readers to the publicly available GHDx documentation, we believe the revised text improves transparency regarding data handling and imputation.

**Comment 5**: Some sections under discussion would benefit from clearer paragraph structure. Ideally, paragraphs should be 4–7 sentences long, with each paragraph focused on one main idea.

Answer 5:We appreciate this helpful comment on manuscript organization. In response, we carefully restructured the Discussion section to improve clarity and readability. Paragraphs were reorganized so that each focuses on a single central theme—such as sex-related differences, national differences in peak age, age-related physiological mechanisms, lifestyle and psychosocial factors, and implications for R-GERD management.

We ensured that paragraph lengths generally fall within the recommended range of 4–7 sentences and that transitions between paragraphs follow a logical progression from descriptive findings to mechanistic interpretation and clinical or public health implications. We believe these revisions have substantially improved the coherence and readability of the Discussion.(Page 14 and 17, Line 307-392)

**Comment 6**: There’s an opportunity to better integrate recent literature, especially by comparing this study’s findings to earlier GBD, or other large-scale GERD related studies.

Answer 6:We appreciate this insightful comment and fully agree that a more explicit integration of recent literature—particularly prior GBD analyses and other large-scale GERD studies—would strengthen the contextualization and significance of our findings.

In response, we have substantially revised and expanded the Introduction section to better situate our study within the existing body of global and national research. Specifically:

1.Enhanced comparison with earlier GBD studies:
We now explicitly discuss findings from prior GBD iterations (e.g., GBD 2017 and GBD 2019), emphasizing both consistent global trends and the methodological and interpretive limitations of earlier analyses when applied to country-specific contexts such as China. This comparison highlights how updates in GBD 2021—including improved modeling strategies and expanded data sources—provide a more robust foundation for long-term trend analysis.

2.Integration of recent large-scale GERD literature (past 5 years):
We incorporated high-quality epidemiological and population-based studies published within the last five years that examine GERD burden, demographic drivers, and temporal patterns. These studies support the concept that stable or declining age-standardized rates can coexist with increasing absolute disease burden due to population aging and growth, thereby reinforcing the relevance of our decomposition and projection analyses.

3.Clear positioning of the current study’s contribution:
The revised Introduction now more clearly delineates how our work extends previous research by providing a long-term (1990–2021), age- and sex-stratified analysis of GERD burden in China using GBD 2021 data, while directly comparing national trends with global patterns. We also emphasize the added value of employing joinpoint regression, decomposition analysis, and Bayesian age–period–cohort modeling to disentangle demographic and epidemiological drivers—an aspect insufficiently addressed in prior studies.

These revisions improve the logical flow and academic depth of the Introduction, strengthen the linkage between our findings and existing literature, and clarify the novelty and policy relevance of the present study.((Page 3 and 4, Line 57-87) )

**Comment 7:** The keywords list should be edited removing China/global-related terms. Keywords such as “DALY” and “GBD” or “Global Burden of Disease” can be added to the list.

Answer 7:Thank you for this helpful suggestion. We agree that the keywords should more accurately reflect the methodological and thematic focus of the study rather than geographic descriptors.

Accordingly, we have revised the keywords list by removing China- and global-related terms and adding more informative and widely used indexing terms, including “DALYs” and “Global Burden of Disease (GBD)”. These changes improve the precision, discoverability, and indexing of the manuscript while better aligning the keywords with the core analytical framework of the study.

Once again, thank you for your valuable feedback on our research. Your insights not only helped us improve the details of our study but also provided us with precious suggestions for future research directions. We look forward to your further comments.

Sincerely,

[Zexu Tang]
